# Automatic early warning of tail biting in pigs: 3D cameras can detect lowered tail posture before an outbreak

**DOI:** 10.1371/journal.pone.0194524

**Published:** 2018-04-04

**Authors:** Richard B. D’Eath, Mhairi Jack, Agnieszka Futro, Darren Talbot, Qiming Zhu, David Barclay, Emma M. Baxter

**Affiliations:** 1 SRUC, Edinburgh, United Kingdom; 2 Royal (Dick) School of Veterinary Studies, University of Edinburgh, Easter Bush, Midlothian, United Kingdom; 3 Innovent Technology Ltd, Turriff, Aberdeenshire, United Kingdom; Gaziosmanpasa University, TURKEY

## Abstract

Tail biting is a major welfare and economic problem for indoor pig producers worldwide. Low tail posture is an early warning sign which could reduce tail biting unpredictability. Taking a precision livestock farming approach, we used Time-of-flight 3D cameras, processing data with machine vision algorithms, to automate the measurement of pig tail posture. Validation of the 3D algorithm found an accuracy of 73.9% at detecting low vs. not low tails (Sensitivity 88.4%, Specificity 66.8%). Twenty-three groups of 29 pigs per group were reared with intact (not docked) tails under typical commercial conditions over 8 batches. 15 groups had tail biting outbreaks, following which enrichment was added to pens and biters and/or victims were removed and treated. 3D data from outbreak groups showed the proportion of low tail detections increased pre-outbreak and declined post-outbreak. Pre-outbreak, the increase in low tails occurred at an increasing rate over time, and the proportion of low tails was higher one week pre-outbreak (-1) than 2 weeks pre-outbreak (-2). Within each batch, an outbreak and a non-outbreak control group were identified. Outbreak groups had more 3D low tail detections in weeks -1, +1 and +2 than their matched controls. Comparing 3D tail posture and tail injury scoring data, a greater proportion of low tails was associated with more injured pigs. Low tails might indicate more than just tail biting as tail posture varied between groups and over time and the proportion of low tails increased when pigs were moved to a new pen. Our findings demonstrate the potential for a 3D machine vision system to automate tail posture detection and provide early warning of tail biting on farm.

## Introduction

Tail biting remains a persistent and unpredictable problem for pig producers worldwide impacting on domestic pig (*Sus scrofa*) welfare and production [1–5]. Being tail bitten is painful [6] and stressful [7] for pigs. Tail wounds can be a source of infection which spreads systemically [8] resulting in further suffering from the resulting morbidity and mortality. In addition this leads to partial or total carcass condemnation at slaughter resulting in losses of around €1.10 per pig produced [9, 10]. There are also negative impacts on pig growth estimated at €0.59 per pig [10] and considerable on-farm labour and veterinary costs [4]. At slaughter, severe tail lesions (part or total tail loss) affect 1–3% of pigs [1, 9] and detectable tail lesions affect over 70% of pigs [10]. On-farm prevalence is very likely higher than abattoir estimates suggest [11, 12].

Tail docking of piglets is widely used to mitigate the harms of tail biting and it does reduce tail biting damage [11, 13, 14], but is not completely effective. Further, this mutilation is itself a welfare concern [5, 6, 13], is seen as undesirable by consumers and its routine use is now banned in the EU (by Council Directive 2008/120/EC). Tail biting can also be reduced (but not completely prevented) by the use of loose material substrates such as straw or wood or by objects hanging in the pen such as knotted ropes which occupy pigs’ behavioural need to root and chew. However, pigs find fresh or novel destructible materials most attractive [15, 16] meaning that materials have to be regularly replenished, which adds expense. Further, there is the technical difficulty that many farms have slatted floors with liquid-slurry systems which cannot cope well with solid materials [3].

Although access to substrates (limited by floor type) is important, several other environmental, system and management factors including stocking density, pigs per stockworker, ammonia levels, temperature, disease status, draughts, nutrition, season, competition for feed and predictability of feed supply are thought to be risk factors for tail biting [1–4, 17, 18], and this lack of a single clear cause makes the problem frustratingly hard to control. The specific trigger for any given tail biting outbreak can vary and is usually unknown, and the uncertainty and unpredictability that many farmers experience can be an important aspect of their motivation to continue tail docking and to seek other solutions [19].

One approach which could reduce this unpredictability is to identify ‘early warning signs’ of tail biting which could be used on farm to identify groups requiring intervention. Two recent reviews have highlighted knowledge of behavioural changes that take place before an outbreak of damaging tail biting [3, 20]. These include: 1) Lowered tail posture- tails are held down rather than up [21–25], 2) Increased activity and/or restlessness [22–24] but see [25], 3) Increased object-directed behaviour [24] and 4) Increased tail biting behaviours: bites which are hard enough to elicit a reaction from the victim [14, 22]. These changes occur at the group level and there is also evidence that certain individual pigs that will become tail biters or victims also show specific changes [26].

In this study, we explore the potential of a ‘precision livestock farming’ approach to tail biting. Precision livestock farming involves the use of modern sensor technologies to detect system, environmental or animal-based indicators of growth, health, behaviour and welfare [27–30].

Sonoda et al [17] suggested that machine vision automated video- based systems could be used in the detection of early warning signs for tail biting. The potential of 3D sensors in farm animal behaviour measurement has been recently discussed [31]. Here we used 3D cameras, and machine vision algorithms to automatically measure tail posture in pigs. Applying this technology to groups of pigs before, during and after tail biting outbreaks, we explore its potential as an automatic early warning system for tail biting.

Our specific aims were to: 1) Validate our 3D tail posture-detecting algorithm (3D tail posture) by comparison with human observers’ assessment of tail posture from video, 2) Establish whether 3D low tail posture increases prior to (and declines after) a tail biting outbreak, 3) Determine whether 3D low tail posture is greater in outbreak than non-outbreak groups and 4) Establish whether 3D low tail posture becomes more frequent with increasing tail injury as assessed by regular clinical inspection of tails.

## Materials and methods

### Ethical considerations

Tail biting is unpredictable, and in order to be certain of having some tail biting outbreaks to study, we did not tail dock pigs and kept them in conditions in which tail biting was expected to occur (i.e. at commercial stocking density in pens with fully slatted floors and limited enrichment). This was considered to be a procedure likely to cause pain, distress or lasting harm under the Animals Scientific Procedures Act (1986) and was regulated by the UK Home Office (Project license number P3850A80D). Ethical approval was also obtained from SRUC’s Animal Experiments Committee (AE 27/2016), and (as a condition from BBSRC for their funding to SRUC) from NC3Rs and BBSRC’s Bioscience for Society Strategy Advisory (BSS) panel. Our primary aim was to collect data on tail posture changes prior to tail biting outbreaks. Pigs were checked at least twice a day by experienced stockworkers or technicians, and once an outbreak was detected, biters were removed and injured pigs were given appropriate veterinary treatment, including analgesia, topical and injected antibiotics, including long lasting antibiotics to reduce the risk of secondary infection. Injured pigs were removed from the pen for recovery in hospital pens if necessary. Pigs remaining in the pen were given enrichment (shredded paper, additional toys and chews—wooden blocks, plastic balls). To prevent further outbreaks, the pen continued to be provided with daily additional enrichment and other strategies were adopted in the event of renewed outbreaks: swapping groups between pens, application of a tail tar (Kerbl Tar Paste, Albert Kerbl GmbH, Germany) and/or provision of molasses blocks (PigLyx—Caltech Crystalyx, Cumbria, UK).

Before the project began, the herd size at the farm was reduced by 25% to free up pen space for hospital pens, and additional deep-straw hospital pens were available and were used to aid recovery in pigs which showed any signs of ill health (136/667 = 20.4% of pigs). Any pigs which were thought to be suffering acutely or had failed to recover with treatment were humanely euthanised by trained staff within one hour. Definition of these endpoints depended on the nature of the ill health, but could include unwillingness to stand, lameness, lethargy and failure to thrive. Euthanasia occurred in 27 cases, and in 12 cases pigs were found dead. This mortality level of 39/667 (5.8%) between weaning and finish is slightly higher than the UK pig industry average figure of 5.0% (https://pork.ahdb.org.uk/prices-stats/costings-herd-performance/rearing-finishing-7-110kg), and probably reflects the higher level of monitoring, and a greater willingness to euthanise pigs to reduce unnecessary suffering. All dead or euthanised pigs were sent for post-mortem examination. Reasons for euthanasia / causes of death included nervous system disease (1), intestinal problems including stasis, necrosis, torsion and bloat (6), heart problems (4), hernia (5), various infections (7), lameness due to swollen joints or fracture (5), lung infections (3), failure to grow and thrive (6) unknown cause (2). Post-mortem examination included bacteriological sensitivity analysis to inform use of the most effective antibiotic for any further cases of secondary infection. It was not possible to determine whether tail biting lesions contributed to any of these deaths for example by being the route of infection. On one occasion the post-mortem report identified tail biting as the suspected cause of intestinal stasis (in the opinion of the post-mortem veterinarian), and ‘tail bitten’ was noted on two further reports. Our legal and ethical duty of care for the health and welfare of pigs in this study continued beyond the data collection period, and they were checked every day and treated if necessary until they were sent for slaughter (mean ± s.d. = 119.3 ± 3.3 days after the study began at weaning). This included regular veterinary checks and approval from a Named Veterinary Surgeon (as per Home Office regulations) that the animals were fit for slaughter.

### Animals and housing

The subjects of this study were 667 intact-tailed pigs (JSR Genetics Large White x Landrace x Hampshire) of both sexes (entire males and females). They were the progeny of 55 sows, housed in pens (2.38m long x 1.52m wide) equipped with standard farrowing crates (2.23m long x 0.47m wide x 1.05m high). The crate had a solid floor with a slatted drainage panel at the back, which was cleaned daily and the crate was replenished with fresh shredded paper and wood shavings. A commercial lactation diet (ForFarmers NOVA; 15% Crude Protein, 13.75 MJ Digestible Energy kg^-1^) was offered twice daily at 0800h and 1500h and was increased from 1.5kg to approximately 6kg per day according to litter size. The pens had a front creep area with heat lamp. Water was available *ad libitum* for both sows and piglets. Piglets were offered a commercial creep diet (Compound pellet creep feed, ForFarmers VIDA Maxima Piglet starter diet) from 7 days of age. Commercial husbandry procedures performed on the piglets included a 1ml iron supplement given intramuscularly at three days post-partum (Gleptosil, Alstoe Animal Health, York, UK) and vaccination for Porcine Circovirus type 2 (Ingelvac CircoFLEX^®^, Boehringer Ingelheim, UK) at 21 days old.

Piglets were weaned into 23 groups of 29.0 ± 3.0 (mean ± s.d.) at 35 days of age. At this point groups were approximately balanced for sex ratio and average piglet weight. Phasing of farrowing dates meant that there were eight contemporary batches of two or three groups at a time, and data collection took place over a period of 7 months. There were two weaner pens per room, each pen measured 2.5m x 2.5m (0.21–0.25m^2^/pig) and had fully-slatted plastic floors, was equipped with a feeder (2.5m in length), nipple drinkers and basic forms of enrichment. This included two flavoured round plastic enrichment devices (Porcichew, Ketchum, Epsom, UK) suspended on chains. During this experiment additional enrichment was added after the first two batches as tail biting outbreaks were occurring regularly. The enrichment included wooden blocks and plastic balls hanging from the side of the pens. Pigs were given creep feed for the first 5 to 7 days before a commercial weaner-grower diet was mixed in (ForFarmers VIDA Ultima) and provided *ad libitum*. Room temperature was maintained at 30°C for the first few days after weaning before being gradually reduced to 24°C before the pigs were moved to grower accommodation. Artificial lighting was operated on a 8h light:16h dark schedule, but the grower rooms had natural ventilation which let in daylight.

Pigs remained in weaner pens for 26.7 ± 0.4 days, when they were moved in their same groups into grower pens (two pens per room). Grower pens (3.20m width x 3.70 length; 0.40–0.47m^2^ per pig) had fully slatted concrete floors and were equipped with two feeders per pen (1.02m each in length), nipple drinkers and two flavoured round plastic enrichment devices (Porcichew, Ketchum, Epsom, UK) suspended on chains. Rooms were initially at 24°C reducing to 20°C seven days after moving in and thereafter. Pigs were fed *ad libitum* with a commercial grower diet (For Farmers HiGro). Pigs remained in grower pens for 25.4 ± 2.2 days at which point the data collection part of the study ended with them being moved to finisher pens at 87.2 ± 2.1 days of age (52.2 ± 2.1 days on the study).

### Tail injury scoring

From weaning until the end of the grower period, pigs had their tails individually scored by a person entering the pen and closely inspecting them. This was done three times a week, usually on a Monday, Wednesday and Friday. Once a tail biting outbreak had occurred in a group (see below), tail scoring was reduced to twice a week on a Monday and Friday. Four aspects of each pig’s tail were scored according to the scheme shown in Table 1: the severity of tail damage (0 to 4), wound freshness (0 to 5), length of tail missing (0 to 3) and the presence or absence of swelling (0/1).

**Table 1:** Tail injury scoring categories.

Category/Score	Short name	Description
**Tail damage**		
0	No tail damage	
1	Flattened	Tail is not round, appears flattened as though it has been sucked or chewed
2	Red	Tail appears red, or has red marks but no broken skin.
3	Puncture marks or scratches	Distinct scratches or puncture marks are visible, skin is broken
4	Wound	Raw flesh visible, tail has sustained tissue damage
**Wound freshness**		
**0**	No Wound	
**1**	Fresh bite or scratch	Not bleeding or weeping (for Damage Score 3 only)
**2**	Intact scab	
**3**	Broken scab	Older blood, red tissue
**4**	Fresh wound—not bleeding	Weeping or bloodied, blood stuck to tail hair
**5**	Fresh wound—bleeding	Blood dripping from tail wound, splattering the pigs’ rump, pen walls or other pigs.
**Tail length**		
**0**	Full length tail	Still has the fluffy bit of hair on the tail tip
**1**	Shortened tail over half remains	Fleshy tail end, tail shortened, but more than half the tail still remains
**2**	Shortened tail less than half remains	Fleshy tail end, less than half the tail length remains
**3**	Tail stump	Less than 1 cm is left of the tail. The tail end is almost flush with the pig’s rump
**Swelling**		
**0**	Tail not swollen	Tail has normal thickness
**1**	Tail swollen	Tail appears swollen: it is thicker than normal, creases in curves, pronounced or hacked

At each scoring event, each pig was scored for all four of these criteria. Modified after [11, 25, 32, 33].

### Tail biting outbreaks

Pigs were inspected at least twice daily with detailed observations during the morning checks. All pigs were checked for signs of tail damage, ill health or lameness with care to ensure all pigs were up and moving around during inspection. Occurrence of a tail biting outbreak was determined based on pen-side observations using only the information usually available to a farmer, and not the information available from our detailed tail scoring. A tail biting outbreak was considered to have occurred once any of the following three criteria was met: 1) when at least three pigs in a pen have fresh tail wounds (see Table 1 - wound freshness score of four or higher) with visibility from outside the pen, or 2) at least one pig with a currently bleeding wound (wound freshness score five) which is obviously seen by dripping blood or splattering, or 3) where there is obvious tail biting behaviour which is causing tail damage, not just ‘manipulating tail’ or ‘tail in mouth’ behaviour [26, 34].

If a tail biting outbreak occurred, protocols were immediately followed to stop the outbreak, prevent further tail biting and safeguard the welfare of the pigs (See Ethical Considerations).

### 3D data collection and processing

Each weaner and grower pen had an IFM O3D301 (https://www.ifm.com/gb/en/product/O3D301) 3D camera orientated to cover around 1/3 of the pen area, located above the feeder pointing vertically down. These cameras use time-of-flight (ToF) technology which sends a pulse of infrared light from an LED 25 times a second, and then records the delay between the pulse and its return to each pixel [31]. Ethernet data cables (Cat 5e) fed the data from each camera to an industrial fan-less PC (http://www.fit-pc.com/web/products/fit-pc4/), connected to a broadband internet connection enabling data download.

Proprietary algorithms produced by Innovent Technology Ltd were used to locate pigs and orient them. For each pig that was present under the camera and standing up, a further algorithm was used to locate the tail and measure its angle relative to the body on a scale of 0 to 90 degrees, where 0 is a tail which is hanging down or tucked against the body so it does not stand out from the curve of the back/rump, and 90 is a tail standing up at 90 degrees. The 3D camera recorded continuously 24 hours a day, and detected tails as often as the system was able. The number of detections is reported in the results. Tail detections cannot be assigned to individual pigs. The system functioned at the group level.

To collate these raw tail angle data into a daily summary of tail posture for each pen, tail angle data were converted into a tail posture score between 0 and 3, where 0° = 0 (low tails), >0° to 30° = 1 (part-raised tails), >30° to 60° = 2 (raised tails) and >60° to 90° = 3 (high tails; referred to subsequently here as 3D 0, 3D 1, 3D 2 and 3D 3). The number of detections in each category were counted, and converted into a proportion of total 3D data for that group and day. Days with fewer than 100 detections were discarded from subsequent analysis, as we considered that data were too sparse to reliably record the overall group level proportion of tail postures.

### 2D video data collection

Each pen was equipped with two 2D video cameras (“Gamet Professional” Sony effio bullet CCTV camera (Gamut, Open 24 seven Ltd, Bristol, UK)); mounted in the ceiling; one capturing the entire pen and one capturing above the feeding area where the 3D camera was also positioned. The two cameras recorded continuously 24 hours per day and video data were stored on the hard drive of a PC-based CCTV system (GeoVision software (GeoVision UK, Letchworth, Herts, UK)).

### 3D data validation by comparison with 2D video

3D and 2D video images were watched simultaneously to validate 3D data ‘by eye’. Three observers viewed data from five, seven and eight groups respectively, selected at random. Data were sampled at intervals between 0800 – 1600h, to obtain between five and 10 observations per hour, on day -1 pre-outbreak (or the same day in matched control pair groups). Where data were missing for technical reasons, the next available frames were used. If data were not available on day -1 outbreak then days -2 and -3 were used. This resulted in a mean (± s.d.) of 45.5 (± 15.5) samples per observer/group, or 911 in total.

For these human observer recordings, tail position was classified as curled, high loose, low loose or tucked down against the body. Curled was defined as a visible loop in the tail, where two parts of the tail overlap, regardless of tail angle. High loose was a non-curled tail that hung at least 45 degrees from the vertical plane of the body. Low loose was a non-curled tail that hung between 0 and 45 degrees from the vertical plane of the body. Tucked was a non-curled tail that was held inwards towards the body. To ensure consistency, the three observers discussed and agreed these classifications before starting. The results of different observers showed agreement over the proportion of pigs in each tail category at a mean (±s.d.) level of 75.2 ± 16.5%.

### Statistical methods

Microsoft Excel was used for organising and summarising data and Genstat 16.1 (VSN International Ltd) was used for analysis. After model fitting, inspection of residual plots was used to confirm the assumptions of normal distribution and heterogeneity of variance without transformation.

To compare injury scoring data (proportion of pigs with 0 damage score) over time between outbreak and contemporary ‘control’ groups with no outbreak, one outbreak and one non-outbreak (control) group were selected from within each batch, based on the amount of 3D data available in the 2 weeks pre-outbreak. The non-outbreak groups’ days were numbered relative to the outbreak of the group they had been paired with. Weeks were coded as -2 (days -14 to -8), -1 (days -7 to -1), +1 (days 0 to 6) and +2 (days 7 to 13). Linear Mixed Models (using REML) were fitted in Genstat. The random model was Batch/Group/Day, and the Fixed model was Week, Outbreak vs Control and their interaction.

Validation of 3D data against human observers’ assessments was analysed using a chi-squared test for association, comparing human observers’ scoring of tucked or not tucked against the algorithm data: 3D 0 or not 3D 0 (3D 1, 2 and 3). Following this validation, it was decided that 3D 0 ‘low tails’ was accurate enough to use in further analysis, but that the other 3D classifications were not.

For 3D tail posture data, all analyses were run using the proportion of detections that day which were 3D 0 ‘low tails’ as the response variate. To begin with, the outbreak groups were analysed to look at changes over time relative to the outbreak day. First, all available pre- and post-outbreak data were analysed, fitting a polynomial regression model with a single curved line for all the data, and then by fitting a line for each group. Second, all available pre- outbreak data were analysed fitting a polynomial regression, followed by fitting a line for each group. Lastly, the effect of week (-2 or -1) on the proportion of 3D 0 low tails for the 14 days prior to an outbreak was tested using ANOVA with Group/Day as the blocking structure. Only 10 of the 16 outbreak groups were included in this analysis as these had at least 2 days of data from each week (week -1, mean ± s.d. = 5.5 ± 2.0 days of data; week -2, 6.3 ± 1.6 days of data; 118 days included in analysis). As well as ANOVA, regression models were also fitted to these data from days -14 to -1. Then, 3D 0 data were compared between outbreak and contemporary ‘control’ groups with no outbreak for the 2 weeks before and after an outbreak, using a mixed model as described above for injury scoring data.

Finally, to determine the relationship between 3D and injury scoring data, a series of Linear Mixed Models were fitted to all of the tail injury scoring data (as the response variable), with separate models with tails low (3D 0) as the explanatory variable (Fixed model). These models always included a random term of batch/group/date.

## Results

### Tail biting outbreaks

There were 15 tail biting outbreaks in the 23 groups (65.2%), occurring between 16 and 41 days (mean ± s.d. = 28.2 ± 9.3) after weaning. Five outbreaks occurred in the weaner pens and 10 in grower pens.

### Tail injury scores over time

**Fig 1:**
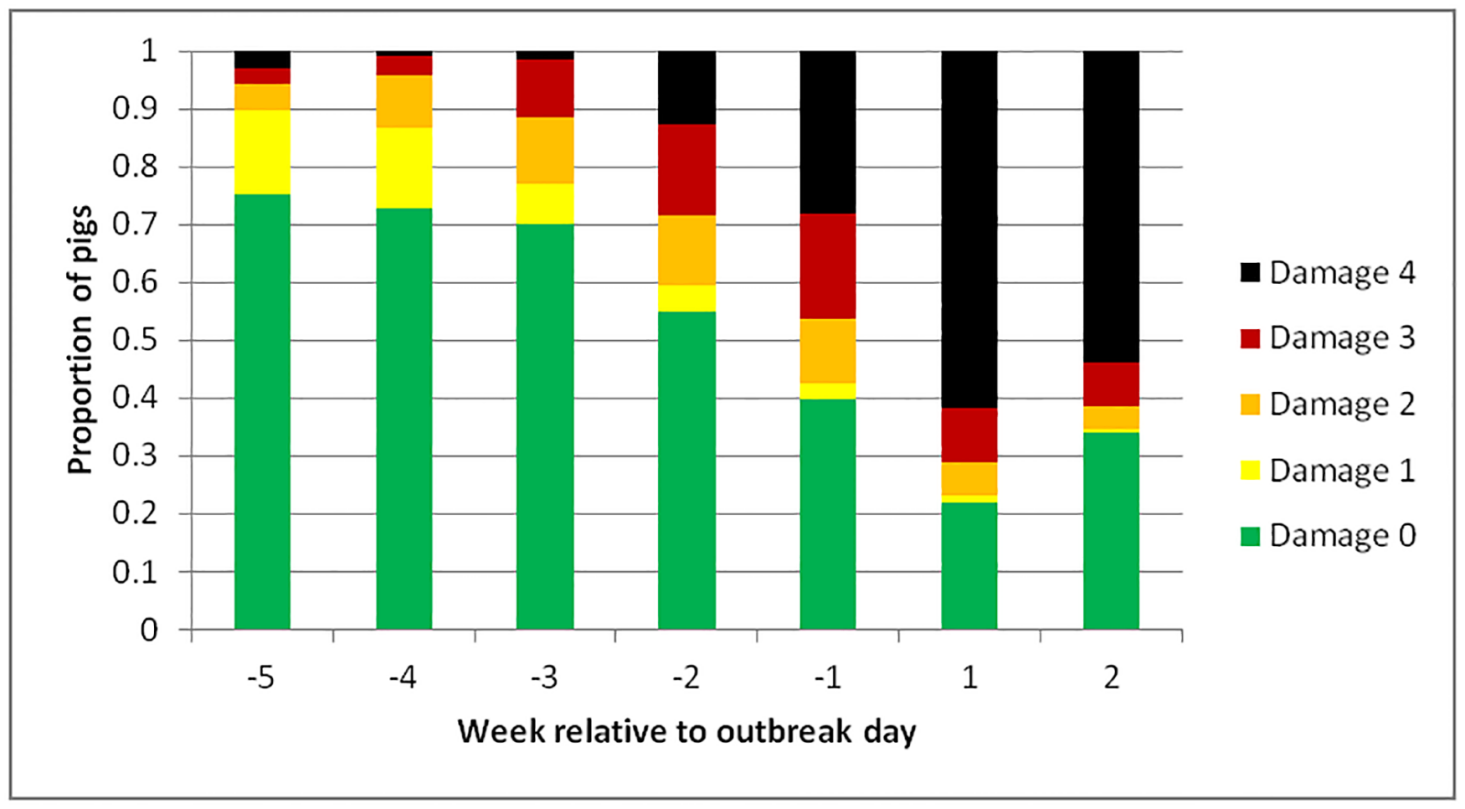
Mean proportion of pigs from the 15 outbreak groups with different tail injury scores (tail damage) in the weeks before and after an outbreak. 0 No damage, 1 Flattened, 2 Red, 3 Bite marks or scratches, 4 Wound.

In total, 12,440 tail injury scores were recorded. For outbreak groups only, the average tail injury score (damage) by week relative to outbreak are shown in Fig 1 and tail injury scores for tail injury freshness, tail length and tail swelling are shown in S1 Fig. Damage and freshness scores began to increase in the weeks prior to an outbreak, while swelling and tail length increased only just before, or after an outbreak. The average proportion of pigs with damage score 0 in non-outbreak groups remained at around 0.6–0.7 over the 2 weeks before and after an outbreak (Week-2 = 0.677 ± 0.064; -1 = 0.663 ± 0.061; +1 = 0.7099 ± 0.0671; +2 = 0.5929 ± 0.0693), while this proportion fell markedly in outbreak groups (Week-2 = 0.556±0.0616; -1 = 0.375 ± 0.061; +1 = 0.198 ± 0.0627; +2 = 0.2065 ± 0.0682). A linear mixed model revealed a significant effect of treatment (F_1,7_ = 24.98, p = 0.002), week (F_3,50_ = 9.53; p < 0.001) and a significant interaction between these (F_3,50_ = 8.01, p < 0.001). LSD tests showed that the difference between control and treatment was significant at p < 0.05 for all weeks except Week -2. Tail injury data for control groups are shown in S2 Fig.

### 3D data collected

1200 days of data (23 groups × mean of 52.2 days) were expected, but due to various technical difficulties, only 962 days were successfully collected, and of these 827 were used for analysis (135 days with fewer than 100 tail detections were discarded from the analysis). In total, 2,152,101 3D tail angle measurements were obtained using the machine vision algorithm, but the number per day was very variable. The greatest number in a day was 20,371, and the mean (± s.d.) was 2237 (± 2746).

Of those tail detections, tail angle was measured at 0° 58.2% of the time (3D 0; 1,251,601 detections), indicating that the tail was hanging too low to be detected relative to the curve of the back. It fell between >0° and 30° 19.3% of the time (3D 1; 416,250 detections), between >30 and 60° 14.9% of the time (3D 2; 321,552 detections) and between >60° and 90° 7.6% of the time (3D 3, 162,698 detections).

### 3D data validation against 2D video

**Fig 2:**
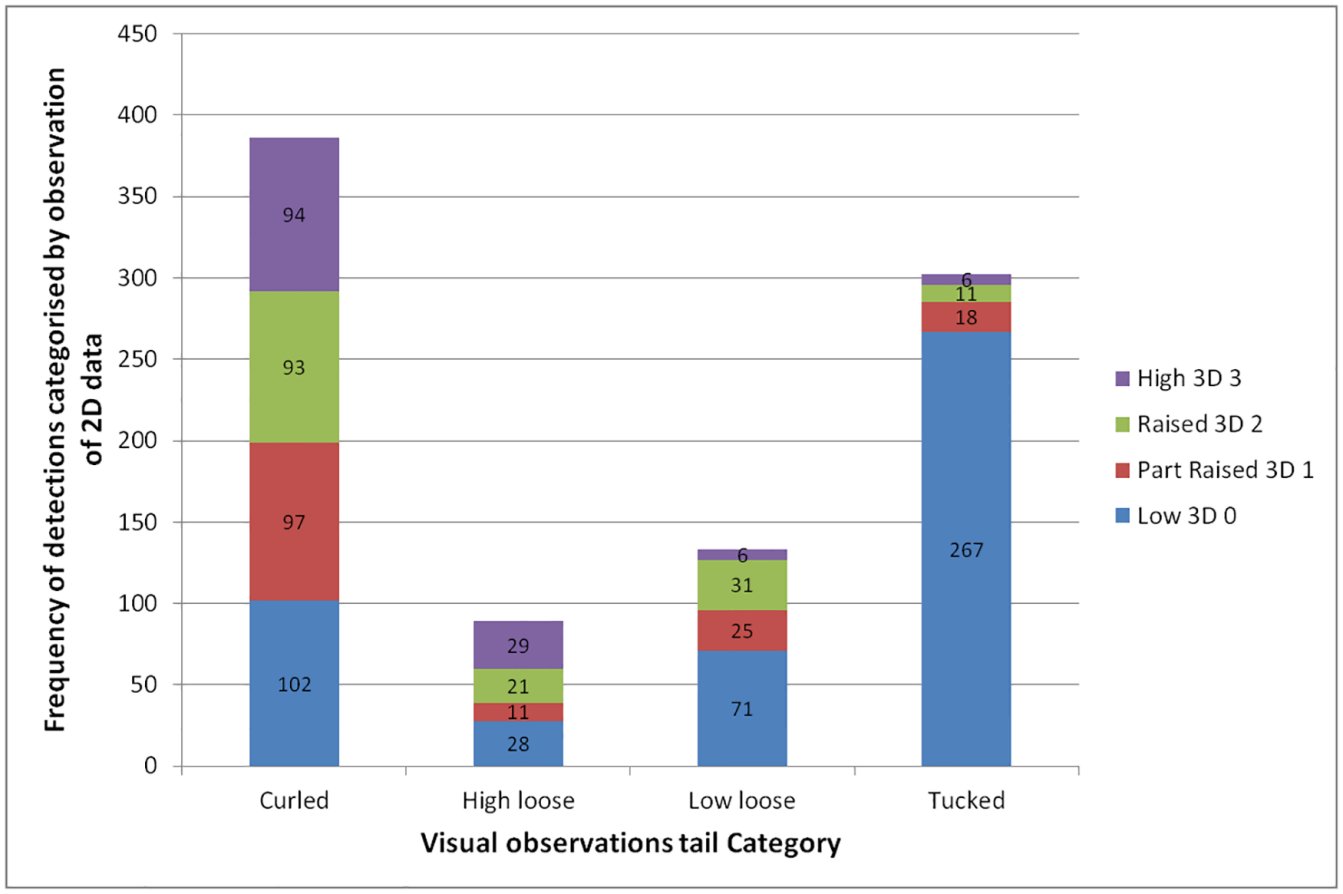
Bar graphs of 3D data validation by a human observer. 926 automatic 3D tail detections were checked by eye and described as curled, high loose, low loose or tucked (‘short tails up’ occurred only 16 times so data are not shown). Data are grouped by the visual observation categories, using different colours for the 3D tail categories. Note that 0° = 3D 0 (low tails), > 0° to 30° = 3D 1 (part-raised tails), > 30°to 60° = 3D 2 (raised tails) and > 60° to 90° = 3D 3 (high tails). Numbers on the bars show the frequencies.

Data from the three observers were similar so they were combined for analysis. In total there were 926 visual validations of the 3D algorithm against 2D video images (Fig 2). The algorithm was good at identifying tails which were tucked low against the body (and quite good at identifying low hanging tails). Tails which were visually identified as tucked (302) were accurately identified by the algorithm as being low (3D 0) 88.4% of the time (267/302 true positive rate; sensitivity). Tails which were visually identified as not being tucked (624) were accurately identified as not being low (3D 1, 2 or 3) 66.8% of the time (417/624 true negative rate; specificity). A chi-squared test on this data showed a significant association between tucked and 3D 0 (χ^2^ = 248.5, p < 0.001). Thus the overall accuracy of the algorithm (correct classifications 684 / total 926) was 73.9%. For hanging low tails, 53.4% (71/133) were classified as 3D 0 by the algorithm. If visual identifications of tucked and hanging low tails are combined and the algorithm is considered correct if it classifies these as 3D 0, then the sensitivity is 77.7%, specificity is 72.3% and accuracy is 74.8%. Of the 474 3D 0 ‘low tail’ observations, these indicated a tucked or low loose tail 71.3% of the time.

Hanging high was correctly identified (as 3D2 or 3D3) 56.2% of the time (50/89). Curly tails were not handled very well by the algorithm despite being quite commonly observed (386/926 = 41.7% of visual checks), being classified fairly evenly across all of the 3D detection categories (Fig 2).

### 3D low tails in outbreak groups pre- and post- outbreak

**Fig 3:**
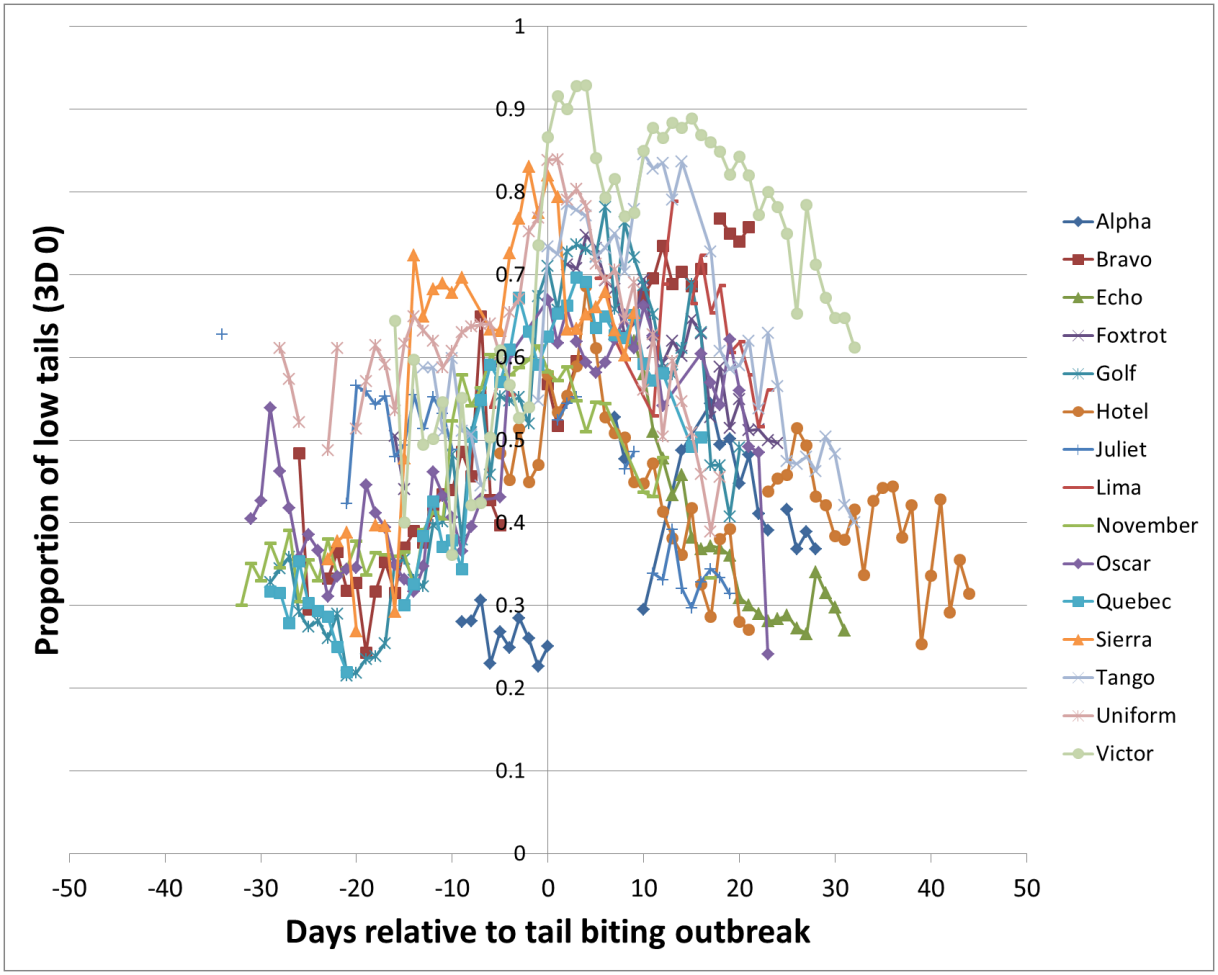
Proportion of 3D tail detections of low tails (3D 0) on the days before and after an outbreak. Data are shown for the 15 outbreak groups, and each line indicates a different group (designated Alpha to Victor).

For the 15 outbreak groups, 3D 0 ‘low tails’ daily proportions were plotted relative to the outbreak day (Fig 3). There were between 25 and 51 (mean ± s.d. = 41.93 ± 7.95) days of data available for each group. A simple polynomial regression model fitted to this data was significant (F_2,543_ = 101.33, p < 0.001; R^2^ = 26.9; Table 2). Linear and Quadratic terms were both significant (Linear term estimate 33.2 × 10^4^, p < 0.001; Quadratic term estimate -2.5 × 10^4^, p < 0.001). A positive linear term indicates that 3D 0 (low tails) increased over time, while a negative quadratic term indicates that the data are curving down (in an inverted U shape; Fig 3), reflecting an increasing proportion of low tails as an outbreak approaches, followed by a decline in low tails after an outbreak, once various mitigation measures are put in place to reduce tail biting.

**Table 2:** Relationship between tail damage scores and 3D low tails data. Data shown are the coefficient of effect (± standard error) for a series of Linear Mixed Models using the proportion of pigs with the various tail injury scores as the response, and proportion of 3D 0 low tails as the predictor (fitting batch/group/date as the random effect model). Data for all groups and all days were used in these models.

Proportion of pigs with Tail Injury Score	3D 0 Low tails
Damage 0	-0.703 ± 0.094***
Damage 1	-0.083 ± 0.032*
Damage 2	-0.210 ± 0.034***
Damage 3	0.100 ± 0.040*
Damage 4	0.831 ± 0.097***
Fresh 0	-1.033 ± 0.082***
Fresh 1,4 or 5	0.400 ± 0.048***
Length 0	NS
Length 1	0.158 ± 0.073*
Swelling 0	-0.197 ± 0.024***

Asterisks are used to indicate the level of significance,

* p < 0.05,

***p < 0.001.

NS indicates that there was no significant relationship.

A second type of regression model was also fitted, in which separate lines were fitted for each group. This was also significant (F_44,501_ = 42.97, p < 0.001) and fitted the data much better (R^2^ = 77.2). Of the 15 groups, 13 had a significant (p < 0.05) linear coefficient. Of these, nine were positive indicating an increase in 3D 0 (low tails) over time, and four were negative. Eleven groups had a significant (p < 0.05) quadratic term. Of these, 10 had negative quadratic coefficients, indicating that the relationship between 3D 0 (low tails) and time takes an inverse-U shape (Fig 3). One group had a positive quadratic term indicating a U shape.

### 3D low tails in outbreak groups pre- outbreak

For the 15 outbreak groups, regression models of the proportion of pigs in the pen with low tail posture (3D 0) on each day, against day were fitted to the pre-outbreak data (data used for this analysis are shown in Fig 3 to the left of the y axis). In this analysis, there were between 1 and 37 days of data available for each group (mean ± s.d. = 20.87 ± 9.87). A simple polynomial regression model fitted to this data was significant (F_2,243_ = 54.3, p < 0.001; R^2^ = 30.3). A positive linear term (193.3 × 10^4^, p < 0.001) indicates that the proportion of low tail pigs increased over days pre-outbreak, and a positive quadratic coefficient (3.6 × 10^4^, p < 0.001) shows that this occurred at an increasing rate. A model fitting a separate line for each group, provided a better overall fit (F_41,204_ = 32.4, p < 0.001; R^2^ = 84.0), eight of these lines had significant positive linear coefficients (six were not significant), and eight had significant positive quadratic coefficients.

### 3D low tails in outbreak groups comparing one week and two weeks pre- outbreak

**Fig 4:**
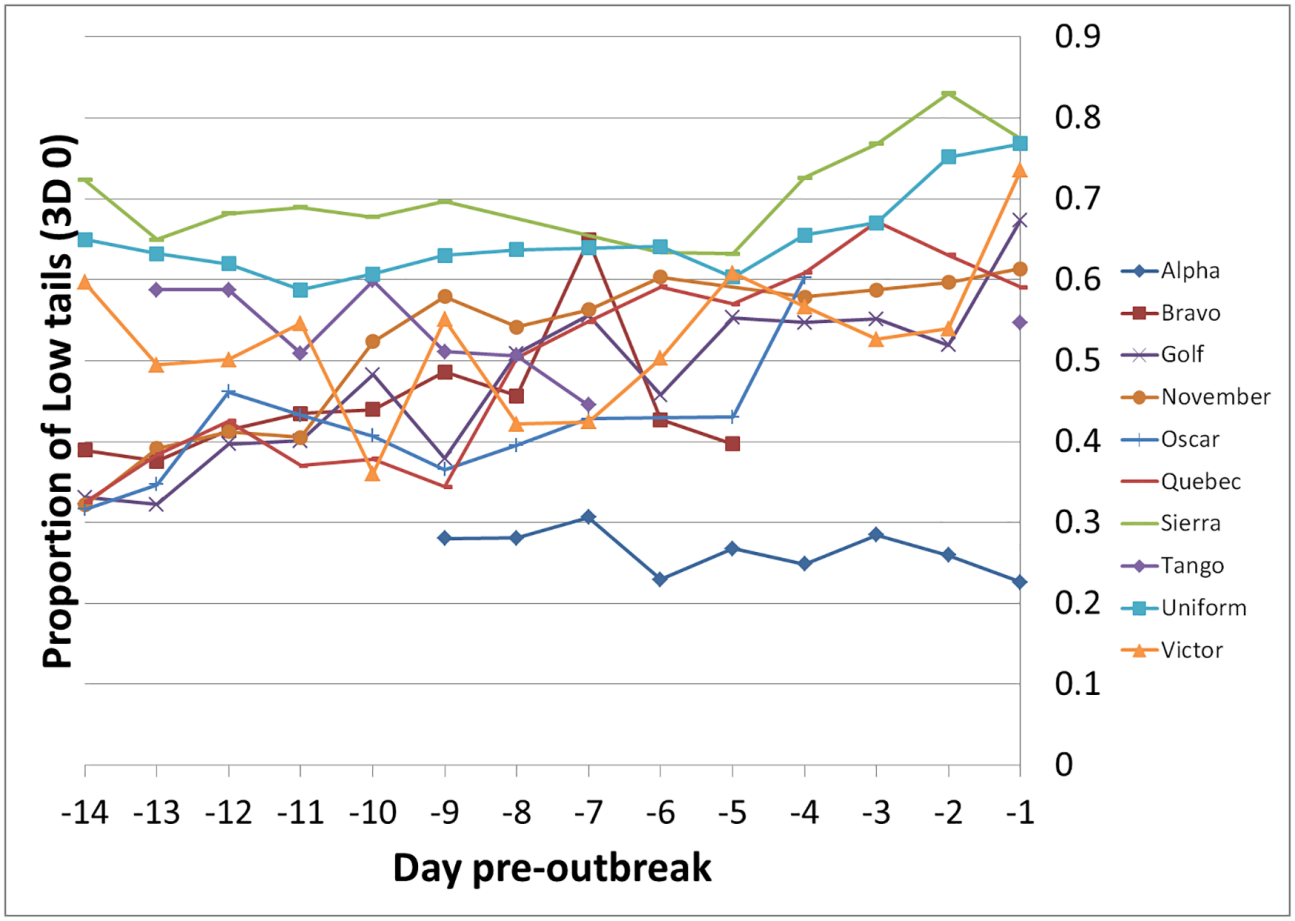
Proportion of 3D low tail detections (3D 0) on the days leading up to an outbreak. Data are shown for the 10 outbreak groups for which there were at least 2 days of data between days -1 and day-7 (week -1) and also 2 days of data between days -8 and -14 (week– 2). Each line shows data for a different group.

For the 10 outbreak groups with sufficient data for this analysis (at least 2 days of data in week -2 and week -1), the proportion of pigs with low tails (3D 0) was higher in week -1 (0.562 ± 0.009) than in week-2 (0.473 ± 0.009, F_1,107_ = 47.5, p < 0.001; Fig 4). This was supported by a significant regression analysis of the same data (F_1,107_ = 67.6, p < 0.001). The positive regression coefficient (0.013 ± 0.002) indicates that the proportion of low tails increased over the 14 days prior to an outbreak. A polynomial regression was also fitted to these data, but the quadratic term was not significant.

### 3D low tails compared between outbreak and control groups

The proportion of low tails (3D 0) for each outbreak vs. control pairing for the 8 batches are shown in S3 Fig. Where data are available, the post-outbreak differences are always clear, and in many cases, outbreak groups appear higher than controls in the days pre-outbreak. The day that pigs were moved from weaner to grower accommodation is also indicated in S3 Fig, and it often appears that this change of pen resulted in an increase in low tails in both control and outbreak groups. Based on this observation, the growth stage was included in analysis of these data. Another notable feature of these graphs is that there are large differences between groups in the baseline proportion of low tails (3D 0).

**Fig 5:**
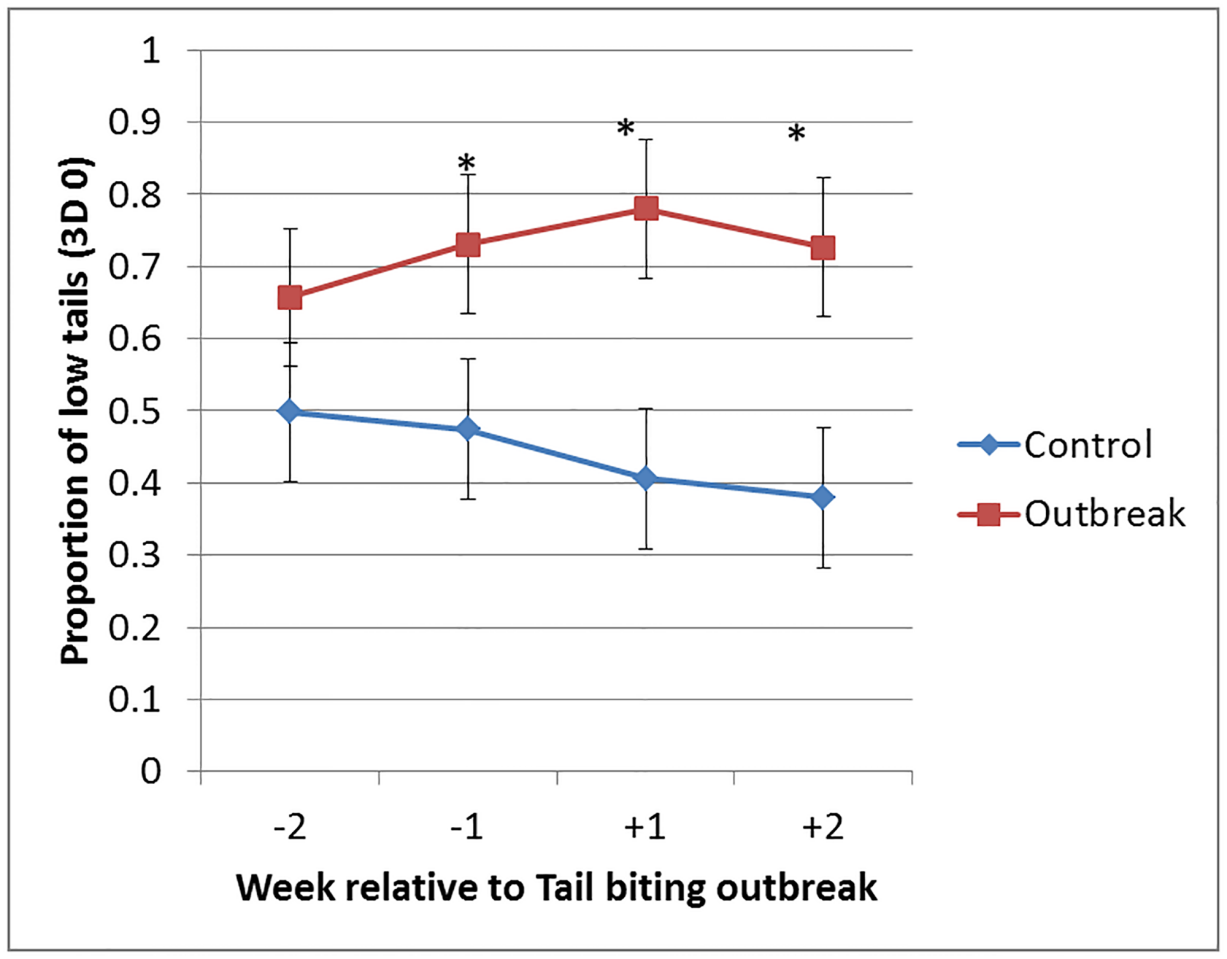
Estimated means (± s.e.) for the proportion of 3D low tail detections (3D 0) during the 2 weeks before and the 2 weeks after a tail biting outbreak. Week -2 was days -14 to -8, week -1 was days -7 to -1, week +1 was days 0 to 6 and week +2 was days 7 to 13. Data were from one outbreak and one control (non-outbreak) group from each of the 8 batches. For control groups, the weeks were assigned by using the outbreak day of their contemporary outbreak group from the same batch. Estimated means were generated from a Linear Mixed Model of Growth Stage (weaner or grower) + Week + Outbreak Vs Control + Week*Outbreak Vs Control, with Batch/Group/Day as the random effects. The * indicates a significant (p<0.05) difference between Outbreak (indicated by red squares) and Control groups (blue diamonds) at the indicated time point (based on Least Significant Difference testing).

A Linear Mixed Model was used to compare outbreak and control groups over weeks -2,-1,+1 and +2 relative to outbreak within each batch. After adjusting for a highly significant effect of growth stage (mean ± s.e. Weaner = 0.53 ± 0.09, Grower = 0.63 ± 0.09; F_1,215_ = 126.5, p < 0.001), the Outbreak pigs showed a higher proportion of low tails than Control pigs (F_1,5_ = 7.47, p = 0.046; Fig 5). Least significant difference tests showed significant differences in low tails between outbreak and control pigs at p < 0.05 during weeks -1, +1 and +2. There was a significant time*treatment interaction (F_3,93_ = 29.5, p < 0.001), low tails increased over time in outbreak but not control groups.

### 3D low tails as a predictor of injury scores

Linear Mixed Models were used to determine the overall relationship between 3D tail posture and tail injury across all groups (including outbreak and control) and days. The negative coefficients of effect in Table 2 show that when there are fewer low tails (3D 0) there are many pigs with uninjured tails (Damage 0, Fresh 0, Swelling 0) or slightly injured tails (Damage 1 or 2). In contrast, positive coefficients of effect in Table 2 indicate that more low tails (3D 0) were predictive of a greater proportion of damaged tails (Damage score 3 or 4), freshly injured tails (Freshness score 1,4 or 5) or reduced tail length (Length 1).

## Discussion

Tail biting is to some degree unpredictable making it difficult to study [3], but the risk factors are known. We were able to induce tail biting in 65% of groups, by keeping pigs with intact tails on slatted floors with minimal enrichment. The timing of outbreaks still remained variable and unpredictable.

Our first aim was to validate the 3D tail posture-detecting algorithm against human observers’ visual assessment of tail posture. The algorithm performance was not perfect, and there is clearly room for improvement for the most commonly seen category of tail posture—curly tails, as the algorithm allocated these fairly evenly across the 3D tail categories. The algorithm did best with tucked tails, correctly allocating them as 3D 0 low tails 88.4% of the time. It also did fairly well with low loose tails, allocating them as 3D 0 low tails 53.4% of the time. We took the decision that only 3D 0 was reliable enough for further analysis, as this metric indicated a tucked or low loose tail 71.3% of the time, and 58% of all tail detections were low (3D 0). Given the difficulty that the algorithm was having with curly tails, it would be interesting to test it in future with tail-docked pigs which are unable to curl their tails.

Our second aim was to establish whether automatically detected (3D 0) low tail posture changed prior to a tail biting outbreak. Regression analysis showed that the proportion of low tails increased pre-outbreak, and at an increasing rate, declining again after an outbreak. When the two weeks pre-outbreak were compared, there was evidence of an increase in low tails from week -2 to week -1, and over the 14 days. These results provide support for the suggestion that low tail posture does increase pre-outbreak as reported by other authors [21–25] and could be used as an (automated) early warning sign of outbreaks.

Our third aim was to compare 3D tail posture data in outbreak and contemporary non-outbreak (control) groups. For a week pre-outbreak (week -1), and post-outbreak (weeks +1 and +2), low tails were higher in outbreak groups than controls, even after the effect of growth stage was taken into account. This provides further support for the idea of using this technology as an early warning sign of tail biting. These outbreak vs. control differences in low tail posture occurred despite the fact that our control groups were not completely free from tail injury. A method to increase contrast in the degree of tail injury between outbreak and control groups would have been to use high levels of enrichment to reduce tail biting risk for control groups. However, this environmental change might itself have influenced tail posture leading to a confound, and the unpredictability of tail biting and variability between groups may still have resulted in some tail damage and tail biting outbreaks in enriched control pens.

Our fourth aim was to establish whether there was a general relationship between 3D tail posture data and tail injury scores across groups and time points. This analysis ignored the distinction between outbreak and control groups. A series of linear mixed models showed that there were significant relationships: when there were many uninjured pigs, or lightly injured pigs in the group, there were fewer low tail posture detections (3D 0). Greater proportions of low tails were seen when injured pigs were more common in the group. The inverse-U shape of the graph in Fig 3 also reflects this increase in low tails as an outbreak draws closer and the decline in low tails after an outbreak when steps are taken to stop further tail biting so tails can recover.

Some authors [22–24] have found increased activity before tail biting outbreaks [but see 25], so activity has potential as an early warning sign. In principle, the number of 3D tail detections per day could be used as a proxy for activity, since pigs must be standing up under the camera to be detected, but the large variability in detections per day meant that we did not try to analyse this. There are various possible reasons for variability in the number of detections. All cameras were connected to a single PC, so the system may have been stretched to capture data from all pens at once. The number of pens under the cameras which had pigs in could vary. There may have been variation in pig behaviour over days in the amount of tail movements such as tail wagging which may have reduced successful tail detections [35]. Some refinements were made to data capture and the algorithm for tail posture detection over the course of the project, which appeared to result in a reduction in missing data and an increase in detections in later batches, but the number of detections per day still remained variable.

In this study, our aim was to identify early indicators of tail biting which were early relative to the stage at which a farmer would usually recognise tail biting and take action. Our criteria for an outbreak (recognised from outside of the pen) was of three pigs with fresh wounds, or one or more pigs with bleeding tails, or obvious tail biting behaviour causing damage. Our tail scoring data (based on closely observing tails from within the pen) showed gradually increasing signs of tail damage at least 1–2 weeks before this point (Fig 1, S1 Fig). Other authors who have worked in this area used different definitions of an outbreak. Zonderland et al [23] analysed data at an individual pig level, and showed that a pig’s tail was more likely to be held tucked between the legs during the transition from no damage to pinhole bite marks, as well as during transitions from uninjured or bite marked tails to clear tail wounds. Lahrmann et al [25] used a definition of 4 pigs in a pen (of ~30 pigs per pen) having a wound, which could include healing or scabbed wounds. If we had used this definition, it would likely have resulted in calling an outbreak in some pens sooner than we did with our method. The concept of an ‘early’ indicator is clearly relative to the definition used for an outbreak of tail biting. The fact that tail injuries are evident through careful tail scoring well before tail biting outbreaks becoming obvious from outside the pen suggests that if an automated method of detecting tail injuries could be developed (e.g. tail colour or tail temperature), this could also give early warning of outbreaks, perhaps in combination with tail posture.

There was considerable variability in the baseline level of the proportion of low tail detections (3D 0) between groups (Figs 3 and 4, S3 Fig), which was also reflected in the much improved regression model fit when lines for each group were fitted. Thus, a method of predicting tail biting from 3D data which depends on detecting a deviation from each groups’ baseline is likely to be the best approach for detecting tail biting. Inspection of S3 Fig suggests that on occasion, trends in tail posture over time co-vary between contemporary groups, probably independently of tail biting. In particular, the proportion of low tails (3D 0) often increases markedly at the same time in both the control and outbreak groups of a contemporary pair when they are moved to a new pen. There is clearly a lot more that we are yet to find out about what affects this ‘background’ tail posture, which would be useful to know for further refinement of an early warning system. For example, it is possible that tail posture could be a general indicator of the state of arousal or of emotional state in pigs [35, 36], meaning that it could be altered by other physical or social stressors, or disease status rather than being only an indicator of early tail biting. An additional concern is that the multifactorial nature of tail biting risk-factors most likely means that some types of outbreak may be more amenable to early detection than others. If an outbreak was triggered by the stress caused by a drinker, feeder, ventilation or heating failure, this could occur relatively rapidly.

The changes in tail posture and tail injury pre-outbreak in our study were similar to those found by other authors [21–25]. Even in the absence of high-tech approaches, these changes, along with signs of tail injury, could be used as advance warning of tail biting outbreaks by farmers with sufficient time to inspect their pigs closely and often enough. Increased awareness and use of early warning signs could reduce the unpredictability of tail biting, giving pig producers greater confidence to cease tail docking in compliance with the requirements of EU council directive 2008/120/EC to use tail docking as ‘a last resort’.

Our findings provide ‘proof of concept’ for the idea that using 3D video cameras to record tail posture could be developed into an early warning system for tail biting outbreaks. Whether this can be applied in commercial farming will depend on whether a real time predictive system can be successfully designed and on the economic cost vs. benefits of such a system. The cost of 3D cameras is relatively high, but their use would be more economically viable if tail biting prediction were only one aspect of a multifunctional system used to detect other commercially important traits such as pig growth [37], aggressive behaviour [38], or behavioural indicators of ill health [29, 39].

In conclusion, our results show for the first time that using Time-of-flight 3D cameras and machine vision algorithms to detect low tail posture have the potential to provide an automatic early warning sign for tail biting. The tail-posture detection algorithm was accurate enough; the proportion of low tails increased over time pre-outbreak, was greater in outbreak groups than control groups, and was associated with increased tail injury. Our study contributes to the rapidly-growing area of ‘precision livestock farming’, using new technologies to inform farm management decisions by providing real-time information on animal growth, health, behaviour and welfare [27–31].

## Supporting information

S1 Fig Mean proportion of pigs from the 15 outbreak groups with different injury scores in the weeks before and after an outbreak. a) Freshness scores (0 No wound, 23 Scab, 145 Fresh), b) Tail length (0 Full length, 1 Shortened, 2 More than half missing, 3 Stump), c) Tail swelling (0 Not swollen, 1 Swollen). Note that Damage scores are shown in Fig 1.
(PDF)

S2 Fig Mean proportion of pigs from the 8 control groups with different injury scores in the weeks before and after an outbreak in their matched-pair within batch outbreak group. a) Damage scores (0 No damage, 1 Flattened, 2 Red, 3 Bite marks or scratches, 4 Wound), b) Freshness scores (0 No wound, 23 Scab, 145 Fresh), c) Tail length (0 Full length, 1 Shortened, 2 More than half missing, 3 Stump), d) Tail swelling (0 Not swollen, 1 Swollen).
(PDF)

S3 Fig Graphs of proportion of low tails (3D 0) over days relative to outbreak for outbreak and control groups within each batch, graphs a)–h) show data for batches 1–8 respectively. The x axis shows days relative to the outbreak for the outbreak group, and the same (calendar) day for the corresponding control group. Outbreak data are indicated by a blue line with diamonds and control data by a red line with squares. The black triangle on the x axis indicates the day on which the pigs were moved from weaner to grower accommodation.
(PDF)

S1 Dataset 3D vs. tail scoring main data. (XLSX)
