# Supplementary figures and images for: Automatic early warning of tail biting in pigs: 3D cameras can detect lowered tail posture before an outbreak
Source: PLoS One. 2018 Apr 4;13(4):e0194524. doi: 10.1371/journal.pone.0194524 (PMC5884497; doi:10.1371/journal.pone.0194524)

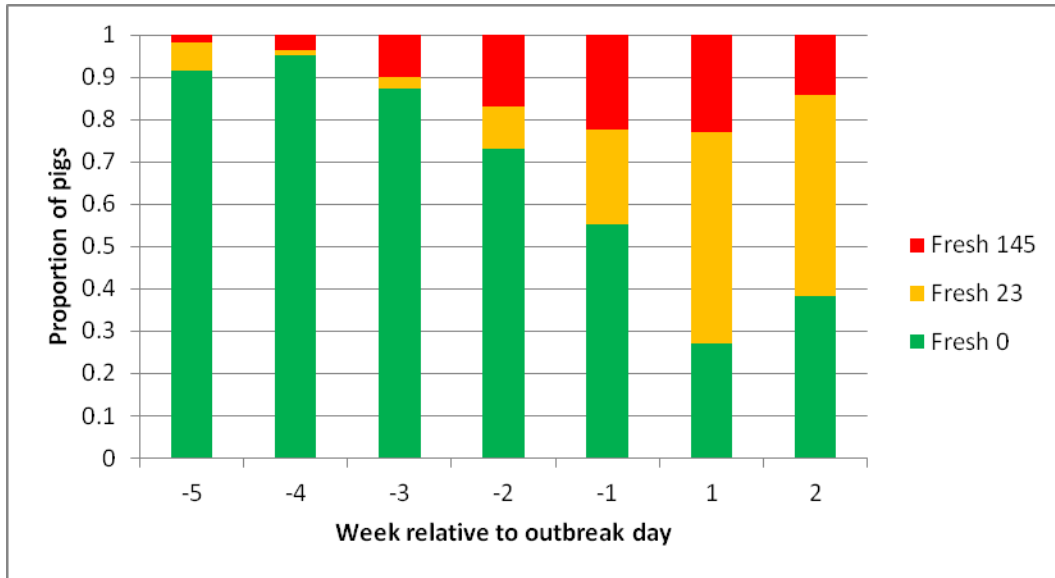

a)

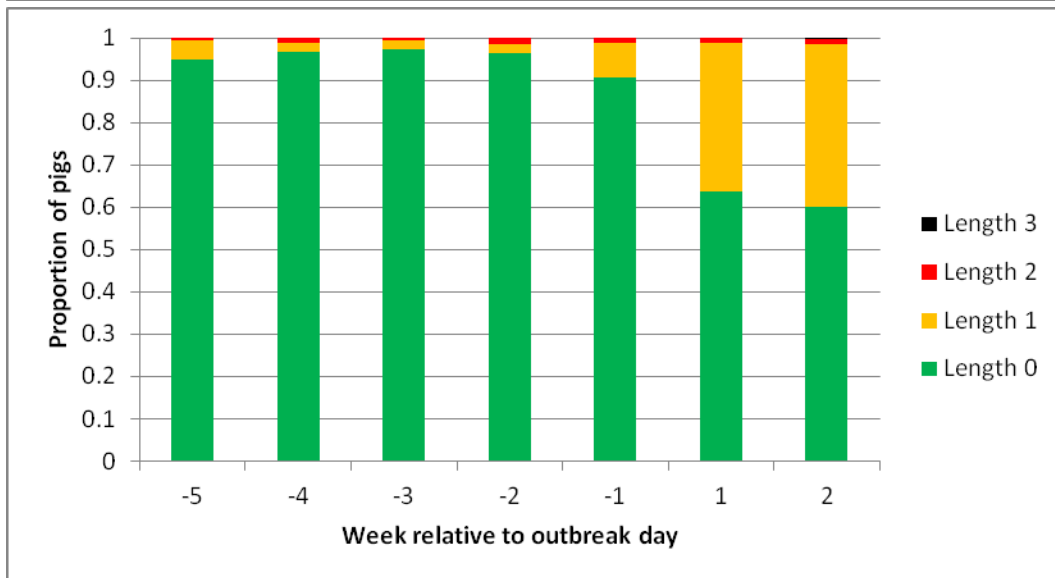

b)

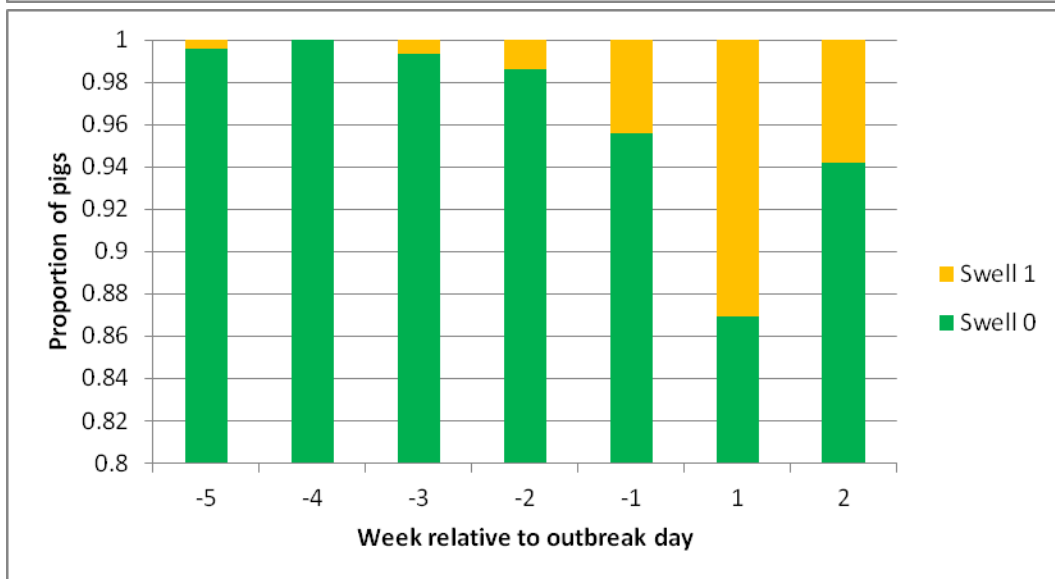

c)

Supplement: S1 Fig — a) Freshness scores (0 No wound, 23 Scab, 145 Fresh), b) Tail length (0 Full length, 1 Shortened, 2 More than half missing, 3 Stump), c) Tail swelling (0 Not swollen, 1 Swollen). Note that Damage scores are shown in Fig 1. (PDF) [file pone.0194524.s001.pdf]

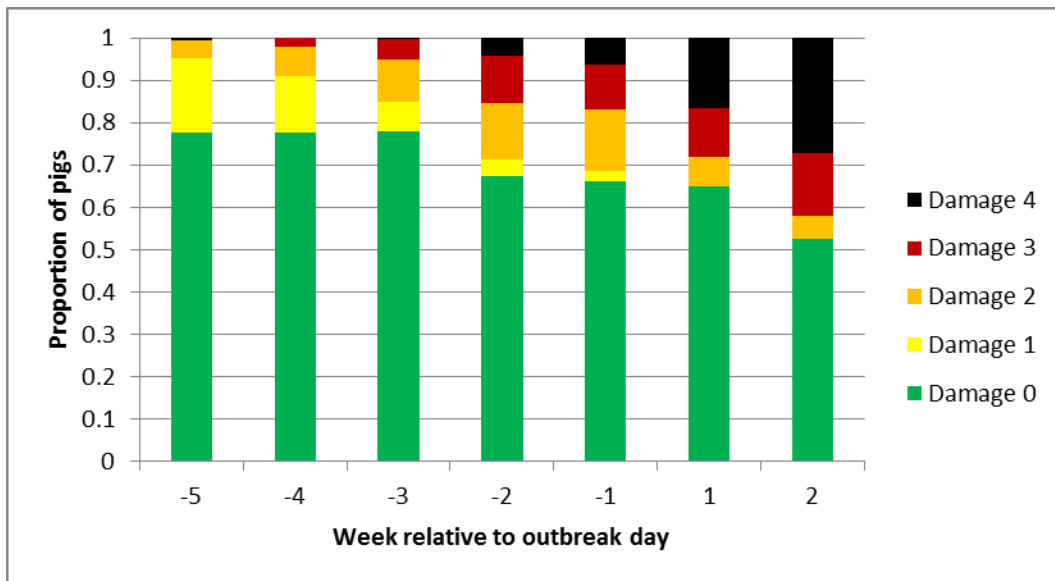

a)

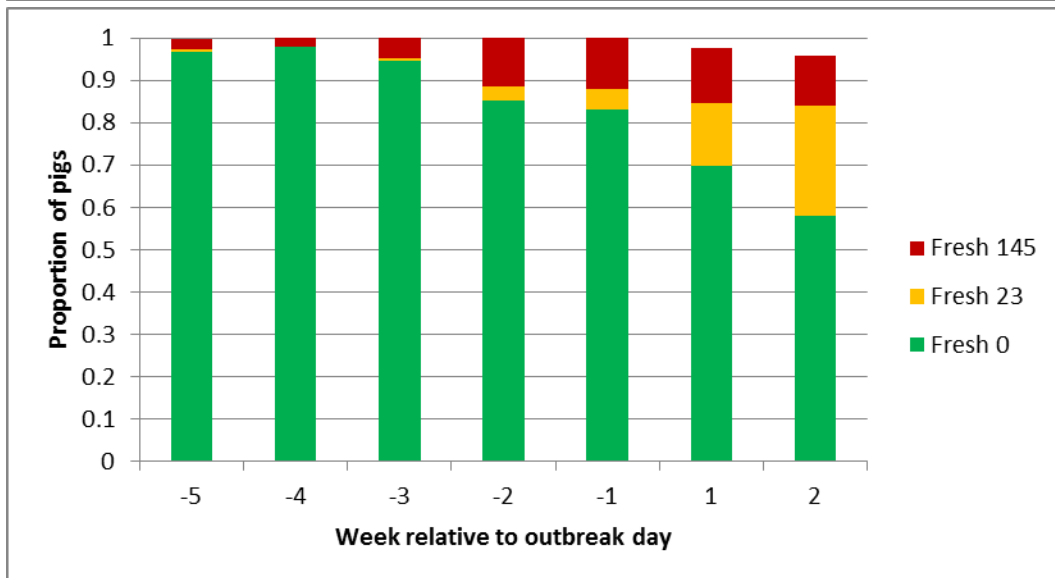

b)

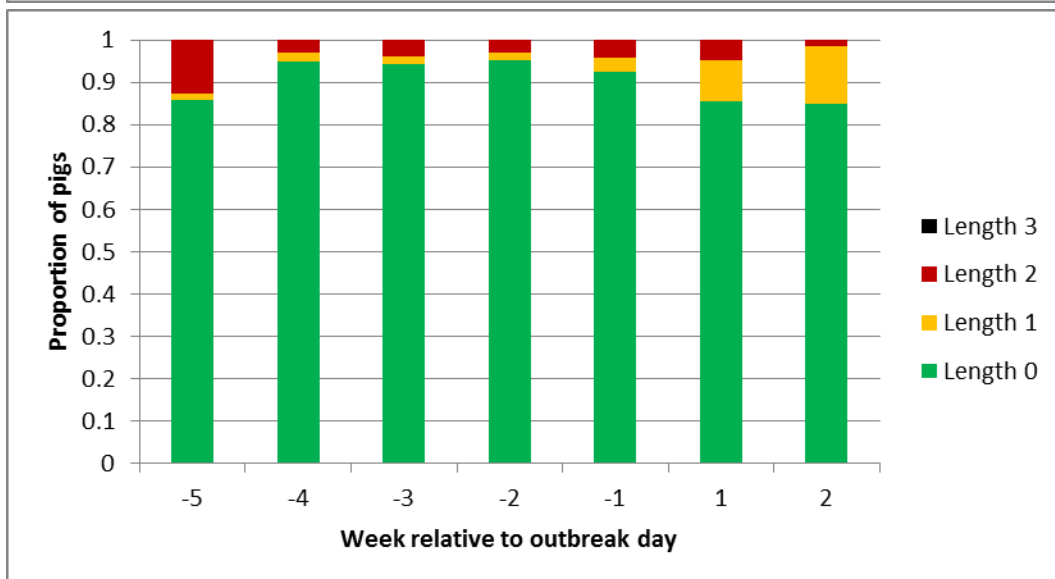

c)

d)

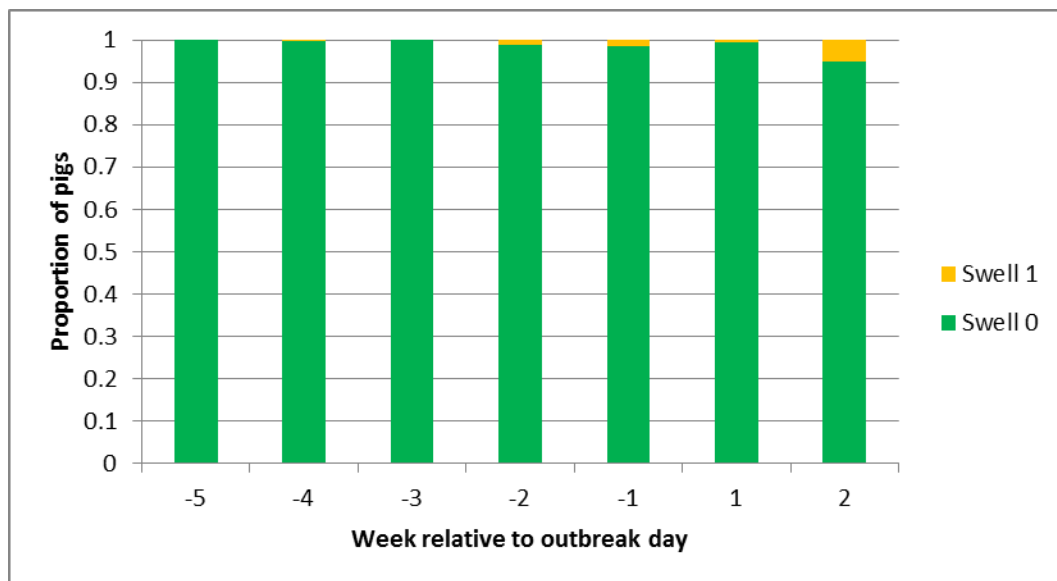

Supplement: S2 Fig — a) Damage scores (0 No damage, 1 Flattened, 2 Red, 3 Bite marks or scratches, 4 Wound), b) Freshness scores (0 No wound, 23 Scab, 145 Fresh), c) Tail length (0 Full length, 1 Shortened, 2 More than half missing, 3 Stump), d) Tail swelling (0 Not swollen, 1 Swollen). (PDF) [file pone.0194524.s002.pdf]

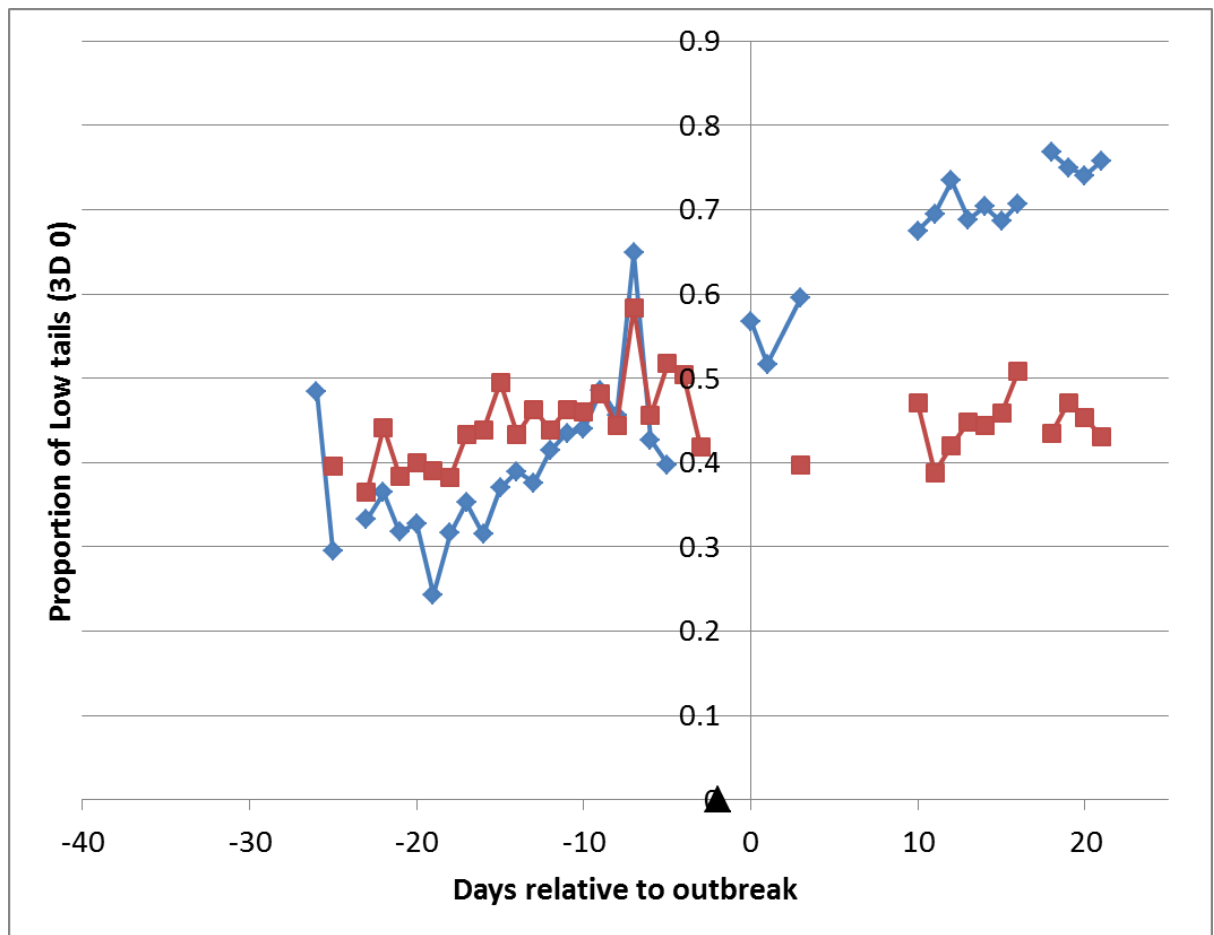

a)

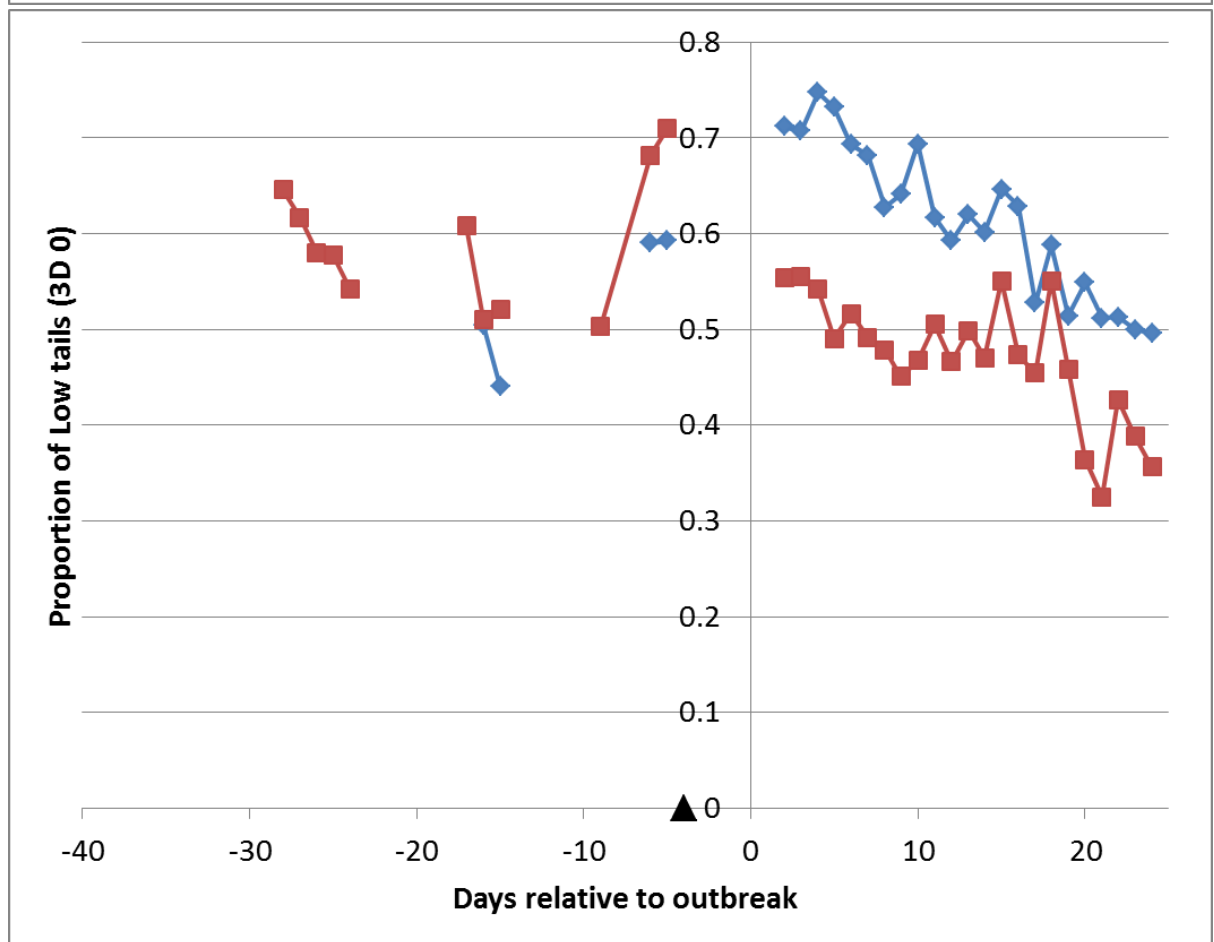

b)

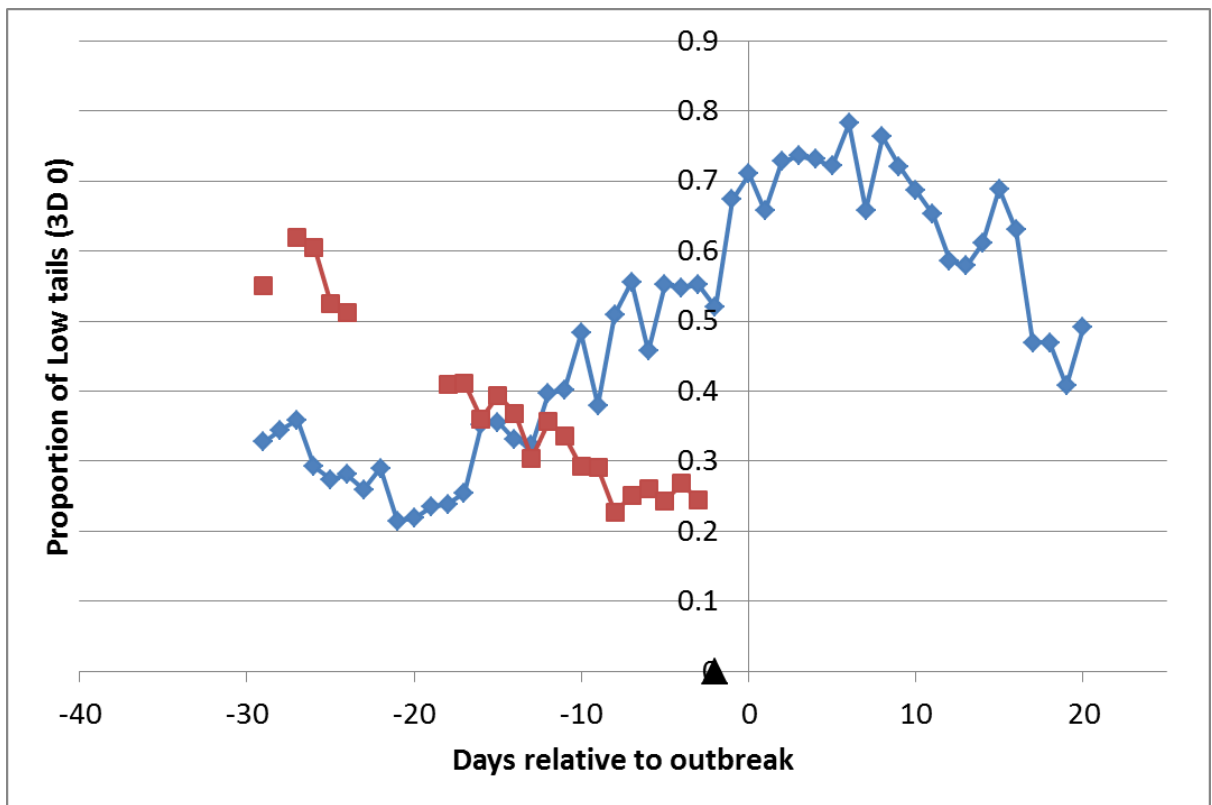

c)

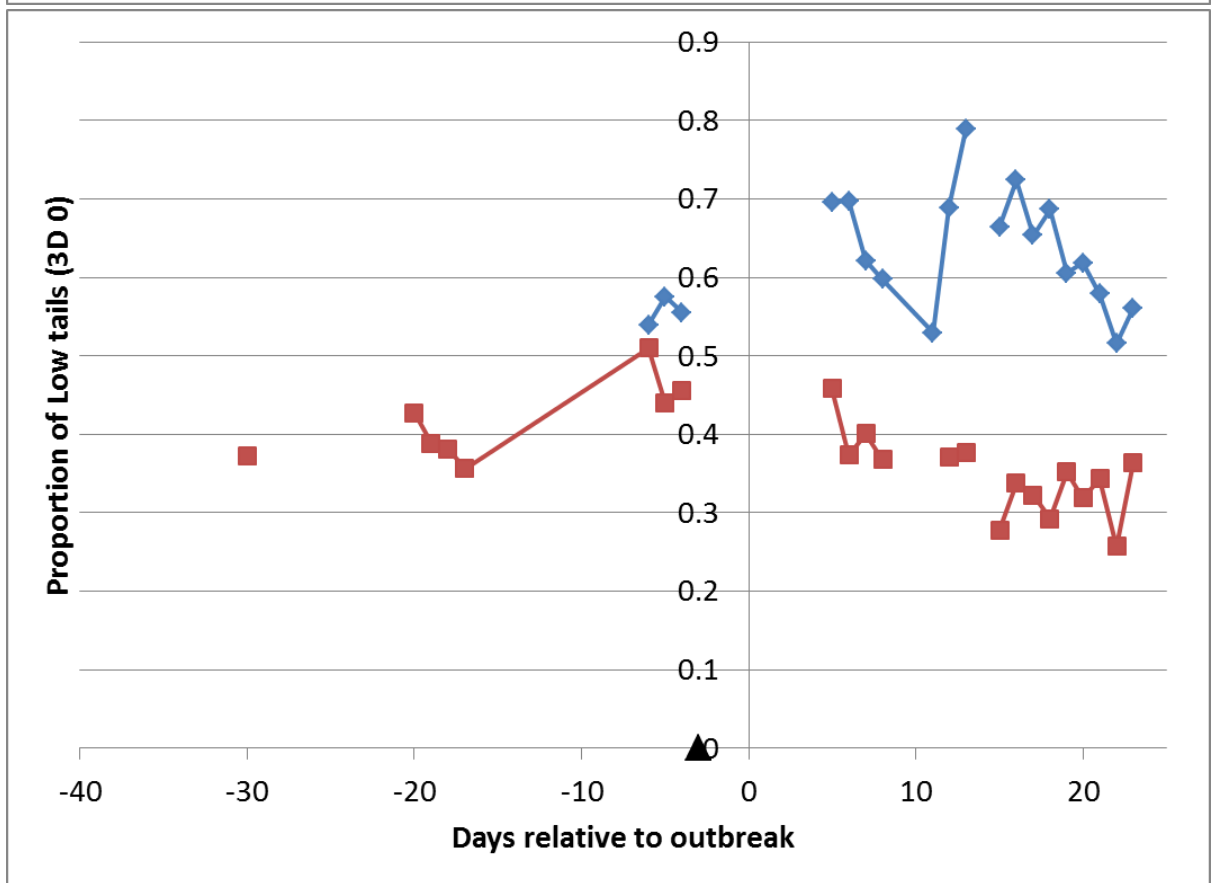

d)

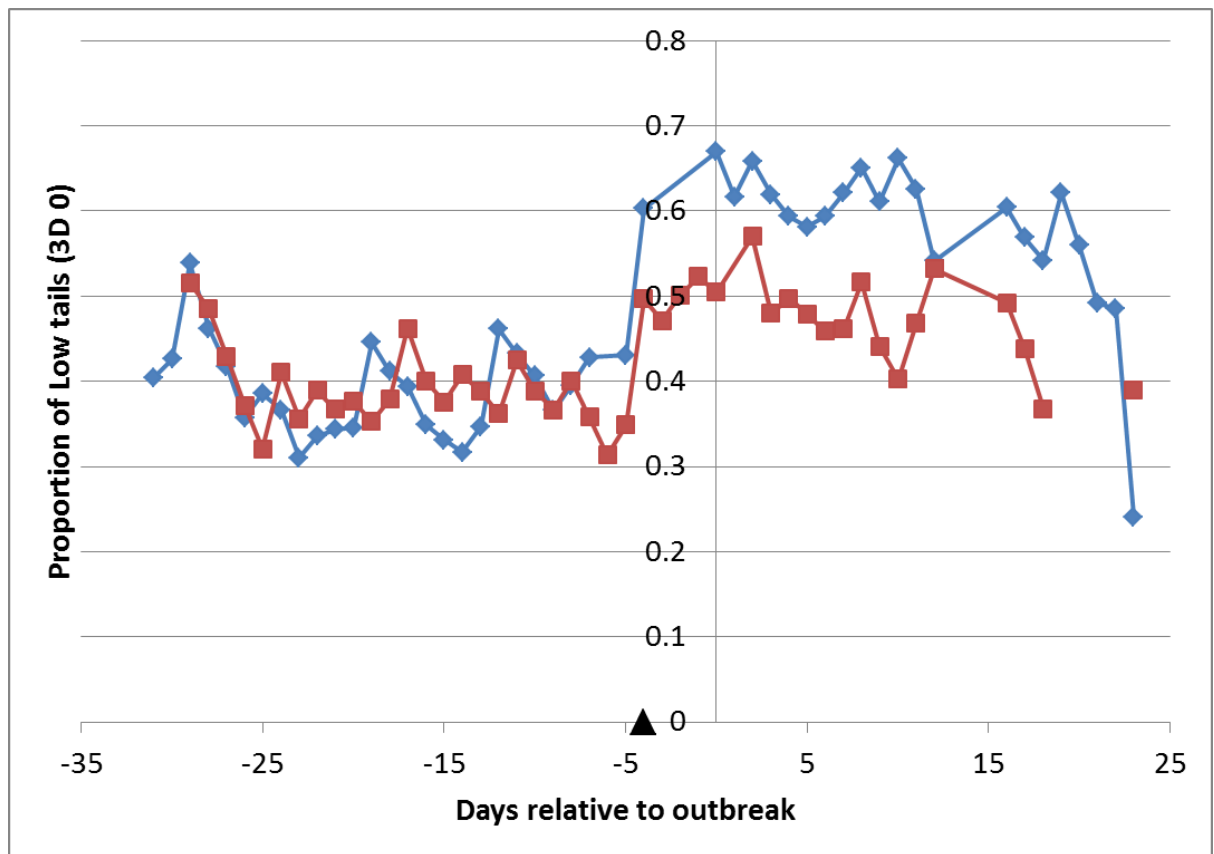

e)

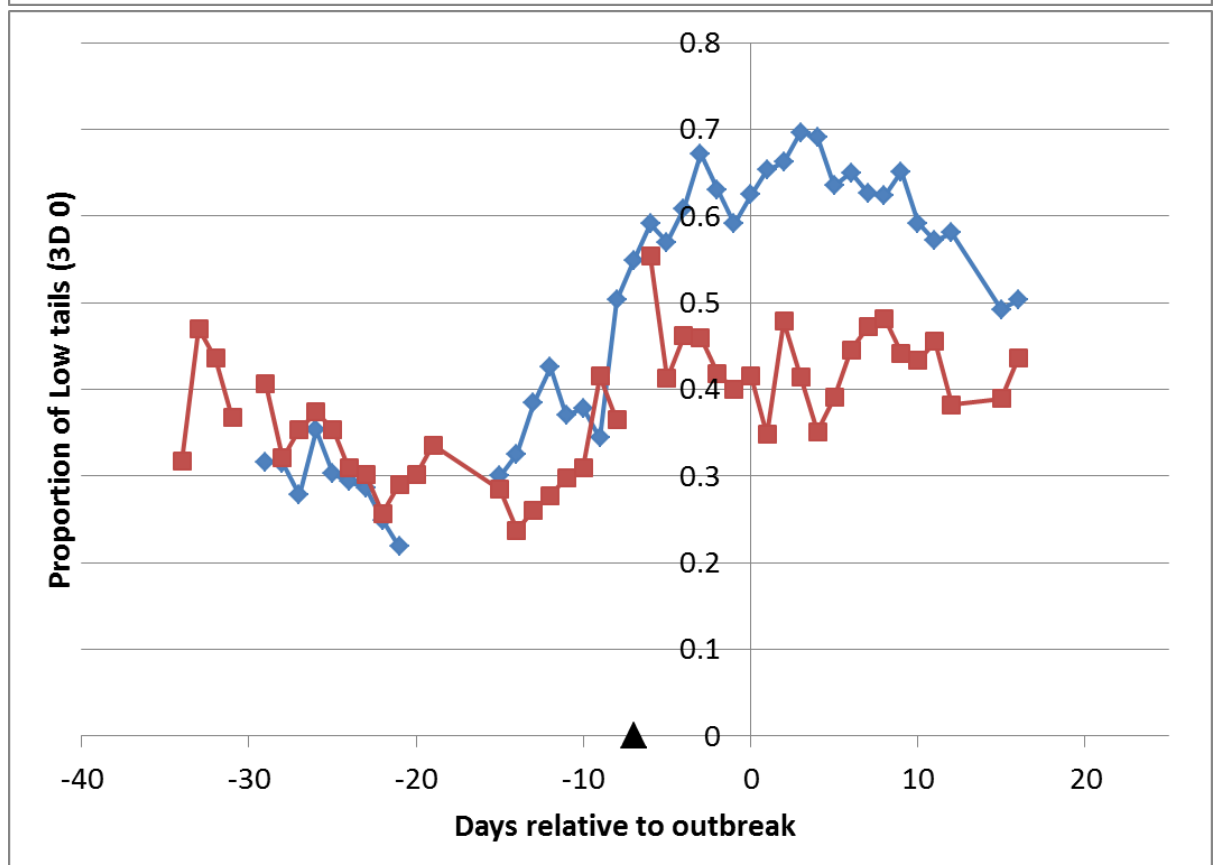

f)

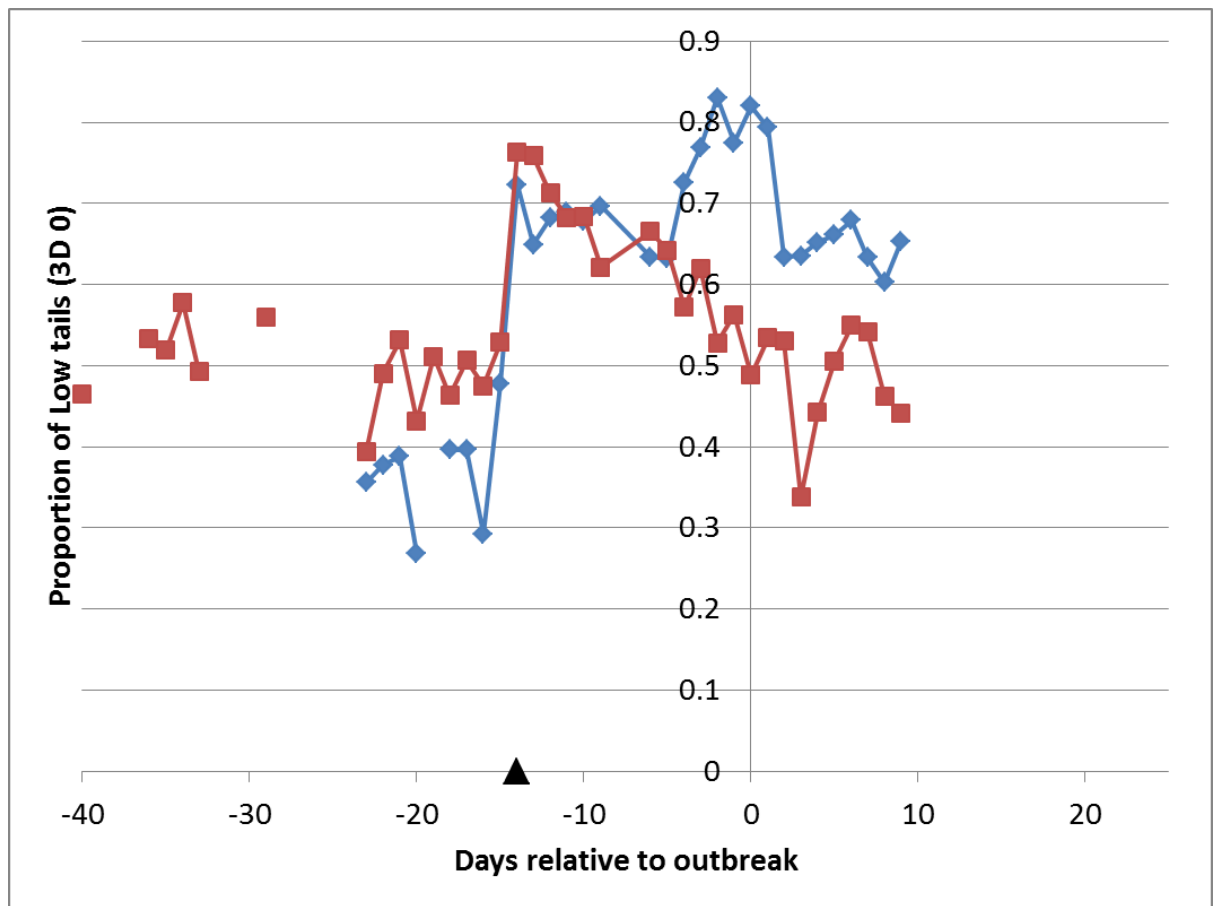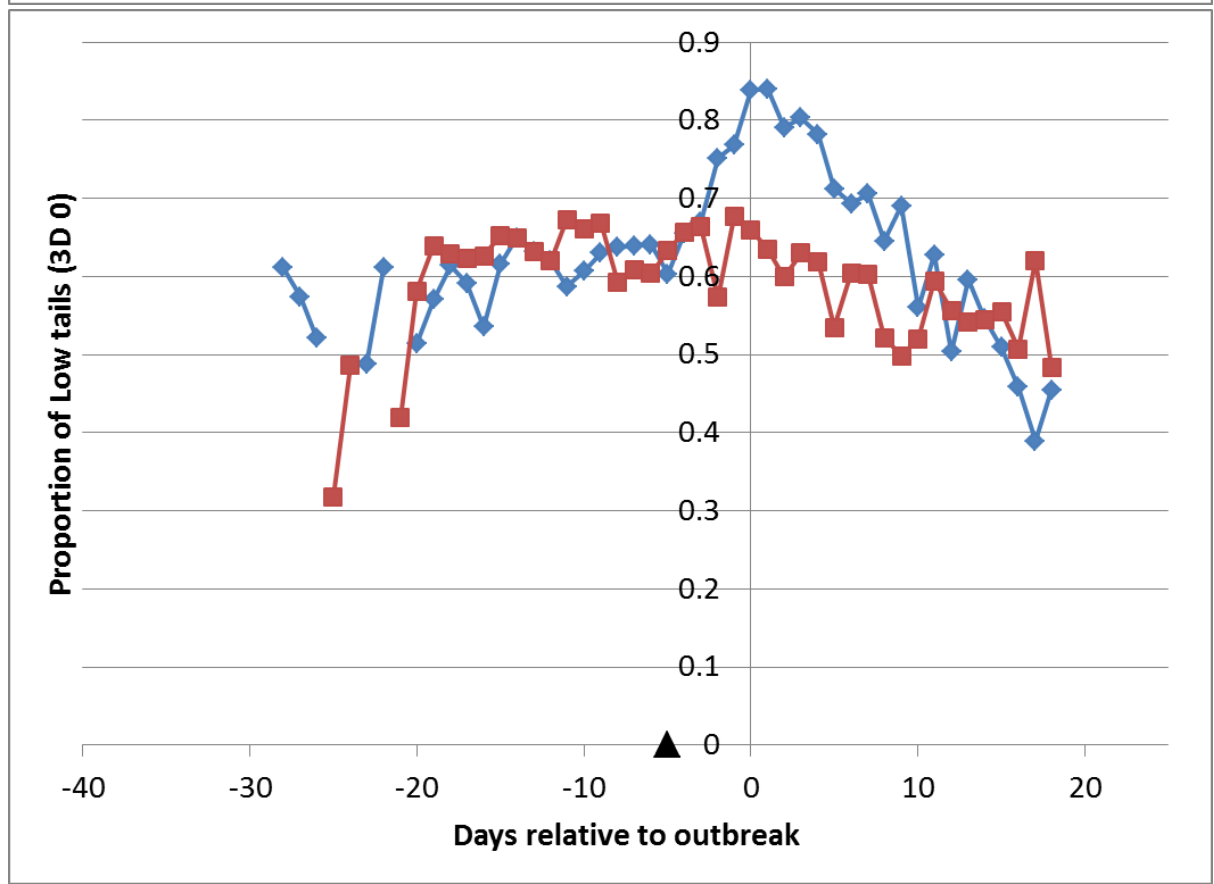

Supplement: S3 Fig — The x axis shows days relative to the outbreak for the outbreak group, and the same (calendar) day for the corresponding control group. Outbreak data are indicated by a blue line with diamonds and control data by a red line with squares. The black triangle on the x axis indicates the day on which the pigs were moved from weaner to grower accommodation. (PDF) [file pone.0194524.s003.pdf]
